# Primary gastrointestinal stromal tumor of the prostate: A case report and literature review

**DOI:** 10.3892/ol.2014.1968

**Published:** 2014-03-12

**Authors:** SULAI LIU, QIUXIA YU, WEIQING HAN, LIN QI, XIONGBING ZU, FUHUA ZENG, YU XIE, JINGSHI LIU

**Affiliations:** 1 Department of Urology, Xiangya Hospital, The Central South University, Changsha, Hunan 410008, P.R. China; 2 Department of Urology, The Affiliated Tumor Hospital of Xiangya Medical School, The Central South University, Changsha, Hunan 410013, P.R. China; 3 Department of Rheumatology, Ningbo Medical Center Li Huili Hospital, Ningbo, Zhejiang 315040, P.R. China; 4 Department of Anesthesia, The Affiliated Tumor Hospital of Xiangya Medical School, The Central South University, Changsha, Hunan 410008, P.R. China

**Keywords:** extragastrointestinal stromal tumors, prostate, c-kit, platelet-derived growth factor receptor-α

## Abstract

Extragastrointestinal stromal tumors (EGISTs) are relatively rare soft tissue neoplasms arising from the extra gastrointestinal tract. The current study presents a case of primary EGIST of the prostate observed in a 55-year-old male. Imaging studies showed a 10×10.5×9.5-cm prostate mass. On histological observation, the mass was separated from the rectum serosa and exhibited a high mitotic count (8/50 high-power fields). The results of immunohistochemical staining showed positive immunoreactivity for cluster of differentiation (CD)117 (c-kit), CD34 and DOG1 in the tumor. On mutation analysis, loss of heterozygosity of the c-kit gene was observed in the prostatic EGIST; however, the platelet-derived growth factor receptor-α (PDGFRA) gene was considered to be normal. Therefore, as EGIST of the prostate is rare, there is a requirement for the confirmation of the diagnosis to be based on immunohistochemistry and mutation analysis (of c-kit and PDGFRA).

## Introduction

Gastrointestinal stromal tumors (GISTs) are the most common tumors of the gastrointestinal (GI) tract. GISTs originate from the interstitial cell of Cajal, an intestinal pacemaker cell in the gut. These cells are known to express the KIT gene (detected as the cluster of differentiation [CD]117 antigen), which is important for distinguishing GIST from other mesenchymal neoplasms (1). In total, approximately two-thirds of GISTs occur in the stomach and approximately one-fifth in the small intestine, occasionally they occur in the rectum, colon and esophagus (2). GISTs that arise primarily outside the GI tract are termed extragastrointestinal stromal tumors (EGISTs). EGISTs are known to arise from various anatomic sites, such as the omentum, mesentery, retroperitoneum and gall bladder. Notably, large, typical, completely differentiated GISTs are rare in the extra GI tract (2). EGISTs that arise in the prostate are extremely rare and only a single case has previously been reported indicating that the prostate is the primary site for GIST (3–7). The current study reports an additional case of a prostatic EGIST, including its presentation, diagnosis, mutation analysis and the type of surgery that was performed, as well as a review of the issues associated with GIST of the prostate.

## Case report

**Figure 1:**
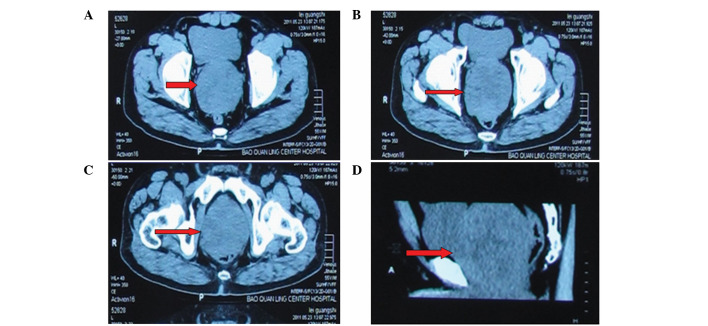
Computed tomography images at four different levels demonstrated a large prostate; size, 10×10.5×9.5 cm. The arrows indicate the tumor.

**Figure 2:**
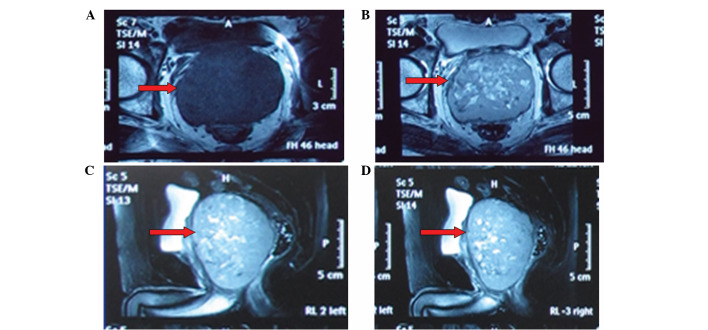
(A) Tumor appeared slightly hypointensive on the axial, unenhanced, T1-weighted magnetic resonance imaging (MRI). (B) Tumor appeared hyperintensive with internal irregular fluid-intense areas on the axial, unenhanced, T2-weighted MRI. (C and D) Tumor was predominantly confined to the prostate without evidence of direct involvement by the adjacent organs. The arrows indicate the tumor.

A 55-year-old male presented to the Department of Urology at the Xiangya Hospital of the Central South University (Changsha, China) with dysuria and urinary frequency that had persisted for approximately six months. The patient’s review of symptoms and medical history were otherwise unremarkable. A digital rectal examination revealed that the prostate was markedly enlarged with a smooth, bulging surface and unusual consistency on palpation. The patient’s prostate-specific antigen (PSA) level was 2.01 ng/ml; all other laboratory values were normal. Computed tomography (CT) showed an enlarged prostate measuring 10×10.5×9.5 cm, but the prostatic capsule was intact (Fig. 1). Magnetic resonance imaging (MRI) showed that the tumor was slightly hypointensive on the T1-weighted images and hyperintensive on the T2-weighted images, with internal irregular fluid-intense areas (Fig. 2A and B), without evidence of infiltration of the rectum and seminal vesicles. However, due to the location and volume of the tumor, the rectum was severely compressed (Fig. 2C and D). No metastatic focus was observed on the chest X-ray or emission CT examination of the skeleton. The patient underwent a preoperative transperineal biopsy for pathological dialysis. The result was highly indicative of EGIST, and high cellularity, high mitotic count, marked nuclear atypism and necrosis supported the high-risk nature of this tumor. To detect the expression of c-kit (CD117), S-100, desmin, CD34, cytokeratin, smooth muscle actin (SMA), DOG1 and vimentin (Vim) proteins, formalin-fixed and paraffin-embedded tissue sections were immunostained with anti-CD117, -CD34, -DOG1 and -Vim antibodies (1:200; Santa Cruz Biotechnology, Inc., Santa Cruz, CA, USA) or anti-cytokeratin, -SMA, -S-100 and -desmin antibodies (1:100; Abcam, Cambridge, UK). All images were captured using a Nikon E1000 microscopic imaging system (Nikon Corporation, Tokyo, Japan). The results of immunohistochemical staining were positive for CD34, DOG1, Vim and CD117, and negative for cytokeratin, SMA, S-100 protein and desmin.

During surgery, the tumor was circumscribed and no associations with the neighboring organs and structures were identified. The tumor was easily separated from the adjacent structures and no enlarged pelvic lymph nodes were detected.

**Figure 3:**
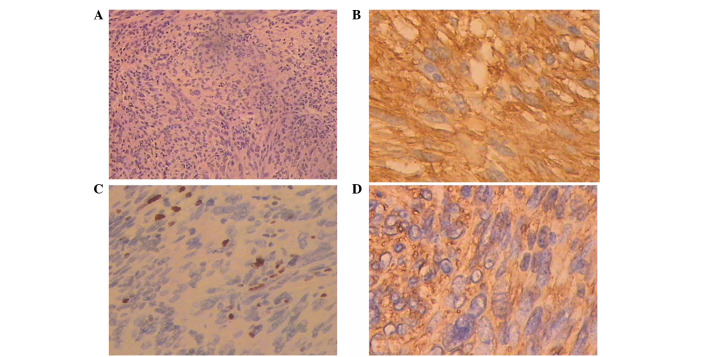
(A) Microscopy (histology) of the resected specimen showed that the tumor predominantly consisted of spindle cells growing in fascicles (stain, H&E; magnification, ×40). Immunohistochemical examination revealed strong positive staining for (B) cluster of differentiation (CD)117, (C) CD34 and (D) DOG1 (stain, H&E; magnification, ×100).

Microscopically, the tumor consisted of spindle-shaped cells with 8/50 high-power fields (HPFs) of mitotic activity (Fig. 3A) and regions of myxoid degeneration. Immunohistochemistry showed immunoreactivity for CD117, CD34, DOG1 and Vim, however, no immunoreactivity was identified for S-100, desmin or SMA (Fig. 3B–D). In addition, the Ki-67 labeling index was low (<1%).

**Figure 4:**
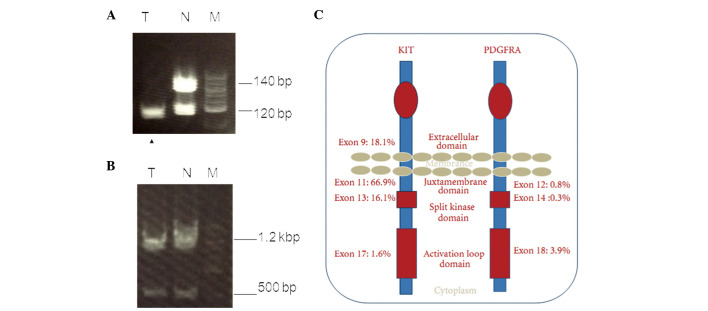
(A) Analysis of microsatellite markers proximal to the c-kit gene. The arrowhead indicates loss of heterozygosity of the c-kit gene. (B) No changes were identified in the PDGFRA gene. (C) Schematic representation of KIT and PDGFRA molecules with location and frequency of mutation (7). Lane N, normal prostate; Lane T, tumor; Lane M, marker; PDGFRA, platelet-derived growth factor receptor-α.

A mutation analysis of exons 9 and 11 of the c-kit gene and those of exons 12 and 18 of the platelet-derived growth factor receptor-α (PDGFRA) gene were examined. Microsatellite markers proximal to the c-kit gene were performed via polymerase chain reaction of the tumor tissue and normal specimens (8). Microsatellite analysis revealed a loss of heterozygosity (LOH) of the c-kit gene in the tumor (Fig. 4A), however, no changes were identified in the PDGFRA gene (Fig. 4B).

The patient had an uneventful postoperative course. As an adjuvant to the postoperative molecular targeted chemotherapy, the patient was treated with an oral administration of 400 mg/day of imatinib (IM). No local recurrence or distant metastasis was observed at the 12-month follow-up.

In accordance with the regulations of the Human Investigation Committee of the Central South University (Changsha, China), written informed consent was obtained from the patient for publication of the current report and any accompanying images.

## Discussion

GIST is a non-epithelial, mesenchymal tumor of the GI tract, occurring predominantly in the stomach and small and large intestines. A mutation in c-kit exons 9, 11, 13 and 17, and PDGFRA exons 12, 14 and 18 is responsible for activation of the gene signaling system, which results in uncontrolled phosphorylation and tissue growth (Fig. 4C) (9). EGISTs are histologically and immunohistochemically comparable with their GI counterparts, however, exhibit an aggressive course, which resembles that of small intestinal stromal tumors. In addition, EGISTs have little or no connection to the abdominal wall or serosal surface of the GI tract (10).

Urologists and urologic pathologists must be particularly aware that certain apparent stromal tumors of the prostate may be rectal GISTs, which involve the prostate in a secondary manner. Previously, Hansel *et al* (11) showed that EGISTs may involve the prostate via a direct extension from the abdominal wall. In addition, Ghobadi *et al* (12) concluded that anorectal GISTs mimic the presentation of prostate cancer. In the current case, the patient did not present any abdominal pain or change in bowel habits. However, clinical and radiological observations did not identify any other primary site of disease or an apparently direct extension from the rectum. The tumor cells diffusely and strongly expressed CD117, CD34 and DOG1 (Fig. 3B–D); therefore, the prostate was considered to be the origin of the tumor.

The clinicopathological features and treatment outcomes of previously described prostatic GISTs, including the present case, are presented in Table I. Dysuria, urinary frequency, hematuria, and pelvic or perineal pain are the common clinical presenting symptoms for prostatic GIST, which has a predilection for adults of >49 years old. GIST is often clinically silent in the initial stages and grows slowly until it reaches a large size. Digital rectal examination and imaging studies often reveal a significantly enlarged prostate. However, the PSA level is almost always within the normal range and previously, one case was identified with multiple liver metastases at diagnosis (4). Immunohistochemistry demonstrated that the mitotic rate was high (5/10 HPFs) in the tumors of the patient in the study by Yinghao *et al* (5) and the present case (8/50 HPFs). However, all cases were considered at high risk of recurrence or metastasis according to the criteria by Yamamoto *et al* (13), and based on tumor size and the histopathological mitotic count (14). However, all four cases demonstrated intense immunoreactivity for CD117 and CD34, and negative staining for S-100. Immunoreactivity for Vim, desmin and SMA was variable in the tumor cells.

In the current case, DOG1, a novel marker originally identified in GIST via gene profiling analysis, exhibited positive immunoreactivity. DOG1 is strongly expressed on the cell surface of GIST and is rarely expressed in other soft tissue tumors; it is also expressed ubiquitously in GIST irrespective of the c-kit or PDGFRA mutation status (15). The reactivity for DOG1 may aid in the diagnosis of EGIST. However, previous studies have established that activating mutations, in KIT and PDGFRA genes, are present in GIST (9,15). In addition, the current study first characterized the gene expression patterns of c-kit and PDGFRA in prostatic EGIST and proposed that the LOH of the c-kit gene may be involved in prostatic EGIST.

Surgery remains the standard treatment for primary resectable EGISTs (5,13). Whenever possible, complete en bloc removal of the tumor and the surrounding organs that are involved is required. The available methods include radical prostatectomy, cystoprostatectomy and total pelvic exenteration. Conventional chemotherapy and radiotherapy are not effective in the treatment of EGISTs and GISTs, whereas IM, a tyrosine kinase inhibitor of c-kit, and PDGFRA as methods of adjuvant therapy, have been proposed as treatment for advanced, unresectable and metastatic GIST. In the evaluation of patients with completely resected primary EGISTs, assessing the risk of recurrence is important for optimal postoperative management. The size, cellularity and mitotic activity of EGISTs have been reported as the most accurate predictors of an adverse outcome (14). Previously, Reith *et al* (10) proposed in a multivariate analysis, that mitotic activity and necrosis exhibit trends towards independent predictive value.

In the present case, the large size (~15 cm), high mitotic counts (8/50 HPFs) and extensive hemorrhagic necrosis of the tumor were the malignant features. Therefore, this tumor belongs to the high-risk group and the results indicated that IM was required.

GISTs exhibit highly variable biological behavior. Although only 10–30% of GISTs are clinically malignant, GISTs harbor a slight malignant potential (16). Therefore, close follow-up is required and must be based upon the risk of recurrence following resection. Generally, high-risk GIST patients relapse early, in a median time of two years following resection (14). Limited data are available to predict the malignant potential of prostatic GIST. However, the available information indicates that EGISTs possess a similar degree of risk as intestinal GISTs (10). The accurate radiological follow-up (abdominal and pelvic CT) is considered to be the approach of choice in the control of EGIST of the prostate.

In conclusion, the current study presents a rare case of EGIST arising from the prostate and the following are proposed: i) It is crucial to note possible adhesion to the rectal wall during pathological examination; ii) immunohistochemistry, using antibodies against CD117 (c-kit) and CD34, is valuable for the diagnosis of EGIST; iii) DOG1 must be included in the routine diagnostic immunohistochemical panel as it allows proper classification of EGIST; iv) mutation analysis (of c-kit and PDGFRA) is potentially significant in the diagnosis and treatment of EGIST; v) IM therapy is recommended for high-risk patients following complete surgical removal of EGIST; and vi) long-term follow-up is necessary.

## Figures and Tables

**Table I:** Comparison of clinicopathological observations, treatment and follow-up data of reported cases of prostatic extragastrointestinal stromal tumors.

First author (ref)	Age, years	Prostate volume, cm	Presentation	PSA, ng/ml	Metastasis	Pathology	Treatment	Follow-up, months
Vander *et al* (4)	49	14.2×9.6×14.0	Urinary retention	1.36	Liver	Mitotic count: abundant; CD117 (+), CD34 (+), SMA (+), S-100 (−) and desmin (−)	IM mesylate	25
Lee *et al* (3)	75	6.7×5.6×5.5	Urinary retention	1.36	(−)	Mitotic count: 15/50 HPFs; CD117 (+), CD34 (+), and disuria Vim (+), SMA (−) and S-100 (−)	Radical prostatectomy	6
Yinghao *et al* (5)	49	8.5×7.0×6.0	Perineum pain	1.1	(−)	Mitotic count: >5/10 HPFs; CD117 (+), CD34 (+), Vim (+), SMA (−), S-100 (−) and desmin (−)	Radical prostatectomy	14
Present case	55	10.0×10.5×9.5	Disuria	2.01	(−)	Mitotic count: 8/50 HPFs; CD117 (+), CD34 (+), Vim (+), SMA (−), S-100 (−), desmin (−) and DOG1 (+)	Radical prostatectomy and IM mesylate	12

PSA, prostate-specific antigen; CD, cluster of differentiation; SMA, smooth muscle actin; IM, imatinib; HPF, high-power field; Vim, vimentin.

## Abbreviations

EGIST: extragastrointestinal stromal tumor
LOH: loss of heterozygosity
GIST: gastrointestinal stromal tumor
IM: imatinib
PDGFRA: platelet-derived growth factor receptor-α
SMA: smooth muscle actin
HPF: high-power fields
GI: gastrointestinal
